# An improved luciferase immunosorbent assay for ultrasensitive detection of antibodies against African swine fever virus

**DOI:** 10.3389/fmicb.2022.1013678

**Published:** 2022-09-29

**Authors:** Qiongjie Wang, Zhancheng Tian, Jifei Yang, Shandian Gao, Junzheng Du, Hongge Zhang, Zhonghui Zhang, Guiquan Guan, Qingli Niu, Hong Yin

**Affiliations:** ^1^African Swine Fever Regional Laboratory of China (Lanzhou), State Key Laboratory of Veterinary Etiological Biology, Key Laboratory of Veterinary Parasitology of Gansu Province, Lanzhou Veterinary Research Institute, Chinese Academy of Agricultural Sciences, Lanzhou, China; ^2^Jiangsu Co-innovation Center for Prevention and Control of Important Animal Infectious Diseases and Zoonoses, State Key Laboratory of Veterinary Etiological Biology Project, Yangzhou, China

**Keywords:** African swine fever virus (ASFV), improved diagnostic tool, luciferase immunosorbent assay (LISA), p35-Luc, sera

## Abstract

African swine fever (ASF), caused by African swine fever virus (ASFV), is a fatal infectious disease of pigs and causes great socioeconomic losses globally. The reliable diagnostic method is critical for prevention and control of the disease. In this study, an improved Luciferase immunosorbent assay (LISA) for detecting ASF was developed using the cell lysates containing ASFV p35 protein fused with a reporter Nano-luciferase (p35-Luc protein). The improved method avoids the complicate procedures of immobilizing the serum samples with protein G in the normal LISA method, and replaced by directly coating the serum samples with carbonate buffer, therefore reduces the productive cost and simplifies the operation procedures. The p35-Luc LISA exhibited high specificity for anti-ASFV sera while no cross-reactions with the sera against other swine viruses. The detection limit of the p35-Luc LISA was shown to be at least four times higher than that of the p35 based indirect ELISA established in our lab. The receiver operating characteristic (ROC) analysis showed the 96.36% relative specificity and 96.97% relative sensitivity of the p35-Luc LISA with the cutoff values of 3.55 as compared to the commercial Ingezim p72-ELISA kit. Furthermore, a total of 248 serum samples were tested by both the p35-Luc LISA and commercial Ingezim p72-ELISA kit, and there was a high degree of agreement (97.6%, kappa = 0.9753) in the performance of the two assays. Collectively, the improved LISA based on the p35-Luc protein could be used as a rapid, ultrasensitive, cost-effective and reliable diagnostic tool for serological survey of ASF in pig farms.

## Introduction

African swine fever (ASF), a highly contagious swine disease, is caused by African swine fever virus (ASFV), a complex enveloped DNA virus. ASFV has a double-stranded DNA molecule of 170 to 190 kb encoding 150-167 viral proteins (Dixon et al., 2013; Liu et al., 2021). ASFV infection causes a high mortality rate with symptoms including high fever, vascular changes, cyanosis of the skin, abdominal pain, and diarrhea in domestic pigs (Zhao et al., 2019; Li et al., 2020). In August 2018, ASFV was firstly introduced into Liaoning Province of China, and was subsequently spread into other provinces or regions, which brought severe threats and challenges to the healthy development of pig industry (Zhao et al., 2019). Until now, none of commercial vaccines or antiviral drugs can be available for ASF control (Li et al., 2021). Therefore, the eradication of ASF in epidemic countries still mostly relied on the rapid and reliable diagnostic procedures (Gallardo et al., 2006).

Due to the occurrence of low virulent mutants, the clinical symptoms caused by ASFV developed from acute mortality at 5-12 days post infection into chronic infection with the non-specific clinical signs (Blome et al., 2013; Gallardo et al., 2015, 2019; C et al., 2018; Pikalo et al., 2019), which increases the difficulty of detecting and controlling the epidemic ASF in farms. Therefore, the reliable diagnostic methods are needed to timely screen and eliminate the infected animals in affected farms (Gallardo et al., 2019). The molecular diagnostic methods are superior in investigating the infected animals with the long-term viremia and high viral load. However, the serological tests maybe have more advantages for recognizing the animals with the weak sporadic viremia (Perez-Filgueira et al., 2006; Cubillos et al., 2013; Gallardo et al., 2019; Qiu et al., 2021).

Previous study have showed that the polyprotein pp62-ELISA was superior to p32-ELISA and p54-ELISA in detecting poorly preserved sera, which allows performance of the diagnosis of ASF without confirmation of the immunoblot test (Gallardo et al., 2006). Thhe polyprotein pp62 produces two major structural proteins, p35 and p15 after proteolytic processing by ASFV S273R (Simon-Mateo et al., 1997; Gallardo et al., 2006), however, which of the protein from the pp62 polyprotein is responsible for the antigenicity is unclear (Gallardo et al., 2006). We found that the p35-ELISA, using the recombinant p35 protein prepared from prokaryotic expression system as antigens, was a reliable diagnostic method in detecting anti-ASFV antibodies (Shi et al., 2021). Nowadays, Luciferase immunosorbent assay (LISA) has been confirmed to be a more rapid and supersensitive method, and exhibit many advantages compared with normal ELISA methods (Wang et al., 2019; Wang T. et al., 2020). Several LISA methods have also been developed and applied successfully in the detection of highly pathogenic viruses including Zika virus (ZIKV), HIV-1, SARS-CoV-2, and Chikungunya virus (Wang et al., 2019; Wang T. et al., 2020; Liang et al., 2021; Li et al., 2022). In the present study, we developed an improved LISA based on the p35-Luc fused protein for detection of anti-ASFV antibodies in clinical practice. The specificity, sensitivity and reproducibility of the p35-Luc LISA were further evaluated and compared with the p35-ELISA and the commercial Ingezim p72-ELISA kit by detecting anti-ASFV antibodies.

## Materials and methods

### Serum samples and reagents

The inactivated ASFV-positive and -negative serum samples were kept and provided by the ASF Regional Laboratory of China (Lanzhou) (Shi et al., 2021), which including inactivated 138 serum samples collected from pig farms in Henan Province after ASFV was introduced into China and 110 serum samples collected from pig farms in Gansu Province before 2015. The sera against four swine pathogens (classical swine fever virus, CSFV; porcine reproductive and respiratory syndrome virus, PRRSV; type-2 porcine circovirus, PCV2; porcine epidemic diarrhea virus, PEDV) were provided by Lanzhou Veterinary Reseach Institute. A bicinchoninic acid (BCA) protein assay kit was purchased from Solarbio Life Sciences, China. INgezim PPA COMPAC (5 plates kit, Lote/Batch: 050819) was purchased from Ingenasa (INGEZIM 11.PPA.K3; Spain). Nano-Glo luciferase kit was purchased from promega (Promega, N1110). HEK293T cell are cultured in DMEM containing 10% FBS at 37°C/5% CO2.

### Identification of p35-Luc proteins

The synthetic full-length p35 gene of ASFV (Pig/HLJ/18strain) (Shi et al., 2021), was amplified with the primers (p35F:5′-GCGAATTCATGGGGAATGACCCGCCGGT-3′, p35R: 5′-CCTCTAGATTAATGGTGATGGTGATGATGCCCCCCTA-3′) including the *Eco*RI and *Xba*I restriction enzyme sites (underlined), and the amplified fragment was subsequently purified and cloned into the secreted luciferase expression vector pNLF-1-N (Promega, USA) by the restriction enzyme sites of *Eco*RI and *Xba*I. The pNLF-1N-p35 recombinant plasmids were verified by sequencing. The amount of 1 μg pNLF-1N-p35 plasmids were transfected into 2 × 10^5^ HEK 293 T cells using lipofectamine™ 2000 transfection reagent (Invitrogen) according to the manufacturer’s instructions. Thirty-six hours after transfection, the 293T cells transfected with the pNLF-1N-p35 recombinant plasmids were lysed in ice-cold RIPA buffer, the cell lysates was then centrifuged at 10,000 g for 5 min at 4 °C to collect the supernatant. The p35-Luc fusion protein was verified via Western blotting with the inactivated ASFV-positive serum kept in our lab (Shi et al., 2021). In addition, the specificity of the p35-Luc fusion protein was determined by detecting of the sera raised against three swine pathogens including PRRSV, CSFV and PCV2 via Western blotting. The supernatants were harvested and stored at −20 °C till use.

### Development of the p35-Luc LISA

**FIGURE 1:**
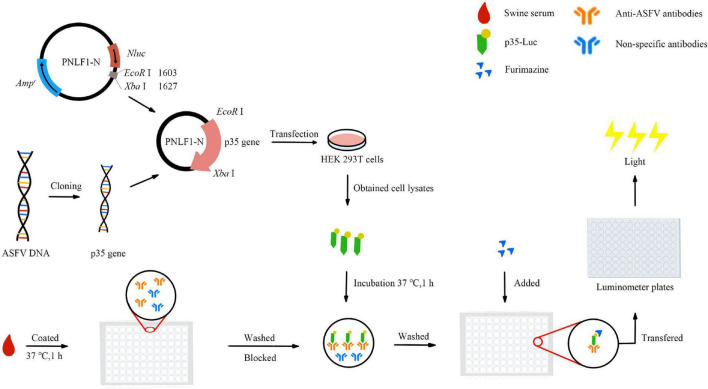
Schematic of the operation procedure of the improved p35-Luc LISA assay.

In the present study, an improved LISA protocol was subsequently developed by using p35-Luc fusion protein, the operation procedures are illustrated in Figure 1. Briefly, 100 μL of diluted testing sera were coated with carbonate buffer (pH 9.6) in 96-well polystyrene microstate ELISA plates (Corning Costar, Corning, NY, USA) for 1 h at 37 °C. After five times washing with PBS containing 0.05% Tween 20 (PBST), each well was incubated with a blocking solution consisting of 1% BSA in PBS for 1 h at 37 °C. Then, the wells were washed five times with PBST. The diluted cell lysates containing the p35-Luc protein were used for detecting inactivated anti-ASFV antibodies. The testing serum samples were incubated with 100 μL of diluted cell lysates containing the p35-Luc protein for 1 h at 37 °C. After five times washing with PBST, 25 μL of the luciferase substrate diluted with buffer (Promega, USA) was added to each well. Then, the mixture was transferred into Costar 96-well flat-bottomed luminometer plates (Corning Costar, Corning, NY, USA), and relative luminescence (RLU) values were determined using a Glo*^Max^* luminometer (Promega, USA) according to the manufacture instructions. Each sample was tested in duplicate. The inactivated ASFV-positive and -standard negative serum controls are set up in each assay.

Under the conditions of different blocking buffer, the 293T cell lysates containing the p35-Luc protein and the inactivated ASFV-positive and -negative serum samples were serially diluted to optimize the protocol of the p35-Luc LISA. According to the optimized p35-Luc LISA protocol, the specificity of this assay was evaluated by detecting the sera raised against four swine pathogens including PRRSV, CSFV, PCV2 and PEDV. Furthermore, 55 ASFV-negative serum samples preserved in our lab collected before 2015 (ASF was not recorded in China) were detected under the optimized p35-Luc LISA protocol, the cutoff value was determined according to the formula: the cutoff value = the average positive-to-negative (P/N) values of negative samples + 3 × standard deviation (s). The tested serum is judged as positive sample when the average P/N values of the tested serum ≥ $\bar{x}$ + 3s, and the tested serum is judged as negative sample when the average P/N values of the test serum < $\bar{x}$ + 2s, and the tested serum is judged as doubtful sample when the average P/N values of the tested serum is located between the cutoff values (Zhang et al., 2017; Shi et al., 2021). The relative specificity and relative sensitivity of the p35-Luc LISA are determined by receiver operating characteristic (ROC) analysis using GraphPad Prism version 8.0 software (San Diego, CA, USA) with 33 inactivated ASFV-positive and 55 -negative serum samples determined by the commercial Ingezim p72-ELISA kit.

### The comparison of the limit of detection (LOD) between the p35-Luc LISA and the p35-ELISA

To directly define the sensitivity of the p35-Luc LISA, the p35-ELISA method established in our lab was used as the control (Shi et al., 2021), an inactivated ASFV-positive and a -negative serum samples were used to analyze and compare the LOD of the both methods. Briefly, the testing serum samples were serially four times diluted starting at 1:100, the values of optical density at 450 nm (OD_450_) RLU for the diluted serum samples were determined via the p35-ELISA and the p35-Luc LISA under the optimized protocol (Shi et al., 2021). Here, the OD values of 0.217 were considered as the cutoff values of the p35-ELISA (Shi et al., 2021) while, the P/N values of 3.55 were setup as the cutoff values of the p35-Luc LISA.

### Stability test

According to the optimized p35-Luc LISA protocol, the same batch of standard inactivated ASFV-positive serum samples with different dilutions was tested with the same batch or different batches of the 293T cell lysates containing the p35-Luc protein. The values of coefficient of variation (*CV*) of the samples within and between batches were calculated to evaluate the intra- and inter-reproducibility of the p35-Luc LISA.

### Reliability test

The performance of the p35-Luc LISA in detecting ASF under the optimized protocol was evaluated through investigating the 128 inactivated ASFV-positive and 120 -negative serum samples determined by the commercial Ingezim p72-ELISA kit. Furthermore, we calculated the kappa values between the two serological tests (p35-Luc LISA and Ingezim p72-ELISA), using the Ingezim p72-ELISA as the reference test to calculate the relative sensitivity and relative specificity of the p35-Luc LISA in the detection of field serum samples.

### Statistical analysis

Statistical analysis was conducted by using GraphPad Prism version 8.0 software (San Diego, CA, USA). The cutoff values, specificity and sensitivity of the p35-Luc LISA were determined through analyzing the receiver operating characteristic (ROC) curve and degree of agreement (kappa value) of a panel of inactivated ASFV-positive and -negative serum samples. All data are shown as means ± standard deviations (SD). The reproducibility of the p35-Luc LISA was evaluated by the coefficient of variation (*CV*).

## Results

### The p35-Luc protein was specifically recognized by anti-ASFV sera

**FIGURE 2:**
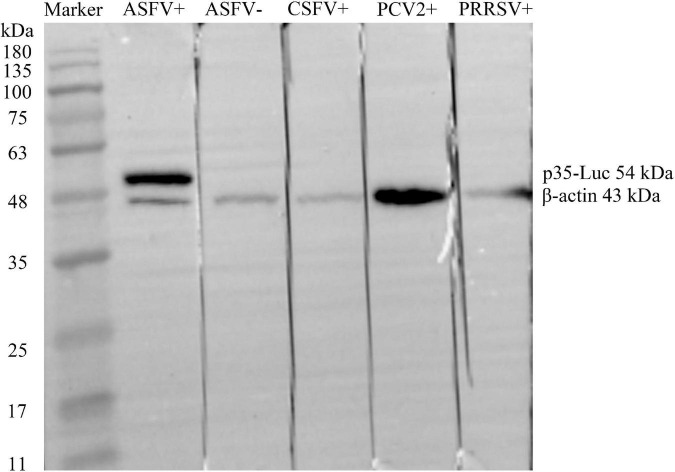
The p35-Luc protein was accurately expressed and was specifically recognized by the inactivated anti-ASFV serum. The expression of p35-Luc was analyzed *via* Western blot with ASFV-positive serum and -negative serum. Lane 1, protein marker; lane 2, ASFV-positive serum; lane 3, ASFV-negative serum; lane 4, CSFV-positive serum; lane 5, PCV2-positive serum; lane 6, PRRSV-positive serum. The β-actin was used as loading control.

As shown in Figure 2, the ASFV p35 gene was cloned into the secreted luciferase expression vector pNLF-1-N (Promega, USA), the 293T cell transfected with the pNLF-1N-p35 recombinant plasmids were lysed and used as crude antigens, Western blotting results showed that a specific band with the predicted molecular weight of 54 kDa was obviously recognized by the inactivated ASFV-positive sera. In addition, no cross reaction was observed with the sera raised against PRRSV, CSFV and PCV2 (Figure 2), implying the reactive specificity of the p35-Luc protein with the inactivated ASFV-positive sera.

### The reactive specificity and optimization of the p35-Luc LISA

**FIGURE 3:**
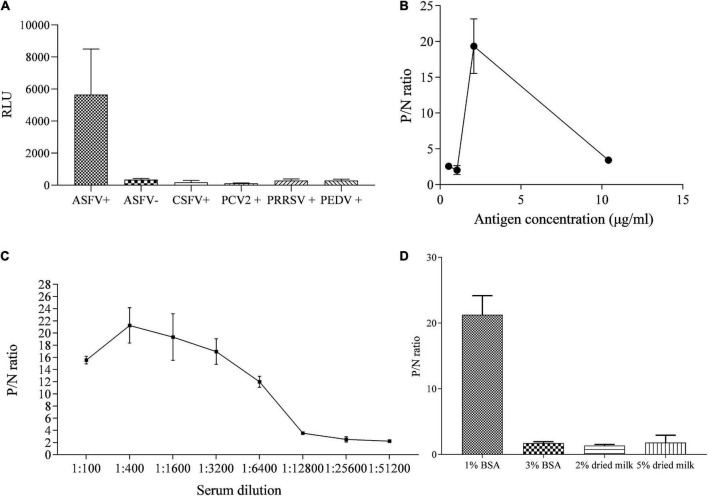
The reactive specificity and optimization of the p35-Luc LISA in detecting ASFV-positive sera. **(A)** The reactive specificity of p35-Luc protein with the ASFV-positive sera was verified *via* an improved Luciferase immunosorbent assay (LISA), the sera raised against four swine pathogens including PCV2, CSFV, PEDV, and PRRSV were used as control serum. P/N values of testing serum samples corresponding to the concentrations of the 293T cell lysates containing the p35-Luc protein **(B)**, the dilution folds of testing serum **(C)**, the blocking buffer **(D)**. Data are shown with means ± SD. Error bars represent the standard deviations from triplicates.

The specificity of p35-Luc protein with the inactivated ASFV-positive serum was further analyzed under the conditions of the cell lysates containing the p35-Luc protein diluted at 1:1000 (250 ng/each reaction) and sera diluted at 1:100, as shown in Figure 3A. The results showed that the luminescence signal targeted on the inactivated ASFV-positive sera was strong but the signal to ASFV-negative sera and the sera raised against four swine viruses (PRRSV, CSFV, PCV2, and PEDV) was very weak, suggesting that the p35-Luc protein can be used as a diagnostic antigen for development of the LISA method for detecting anti-ASFV antibodies.

The differences in the RLU values targeted on the inactivated ASFV-positive and -negative serum samples determined the performance of the LISA method. In the optimized protocol for the p35-Luc LISA, to avoid the difference of transfection efficiency and protein expression of different batches of preparations, the luciferase activities of the diluted crude antigens (1:1000) obtained in the 2 × 10^5^ 293T cells that were transfected with 1 μg pNLF-1N-p35 recombinant plasmids for 36 h were determined, the results showed that the average RLU values is about 4 × 10^6^, therefore crude antigens should be added in each reaction with 4 × 10^6^ RLU for LISA (Figure 3B), all serum samples were diluted to 1:400 in carbonate-bicarbonate buffer (pH 9.6) (Figure 3C), and 1% BSA have the best blocking efficiency as compared to other blocking buffer (Figure 3D), as shown by higher P/N values.

### The cutoff values, specificity and sensitivity of the p35-Luc LISA

**FIGURE 4:**
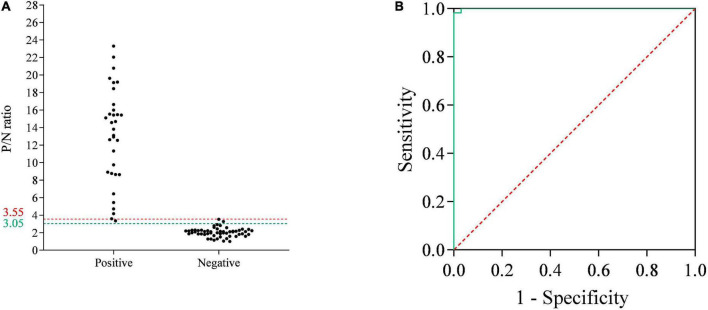
The receiver operating characteristic (ROC) analysis of the p35-Luc LISA. **(A)** The serum samples that are determined as positive or negative by the commercial Ingezim p72-ELISA kit are evaluated by the p35-Luc LISA assay. The two horizontal lines delimit the doubtful p35-Luc LISA results (3.05/3.55). **(B)** ROC curve based on the data obtained.

To determine the cutoff values of the p35-Luc LISA, 33 inactivated ASFV-positive and 55 -negative serum samples, determined by the commercial Ingezim p72-ELISA kit, were used to analyze the cutoff values and the relative specificity and relative sensitivity of the p35-Luc LISA based on the ROC curve. Here, the average P/N values was 2.04, with a standard deviation of 0.50, the tested serum is judged as positive sample when the average P/N values of the tested serum ≥ 3.55, the tested serum is judged as negative sample when the average P/N values of the tested serum < 3.05. According to the above criteria, the one false-positive serum sample and two false-negative serum samples were defined in the p35-Luc LISA (Figure 4A), and these doubtful samples further were validated by the p30-ELISA established in our lab (Shi et al., 2021), the validated results showed the consistency between the p30-ELISA and the commercial Ingezim p72-ELISA. As shown in Figures 4A,B and the p35-Luc LISA conferred 96.36% relative specificity and 96.97% relative sensitivity for the detection of ASFV antibody as compared to the Ingezim p72-ELISA kit.

### The p35-Luc LISA is ultrasensitive than the p35-ELISA for detecting anti-ASFV antibodies

**FIGURE 5:**
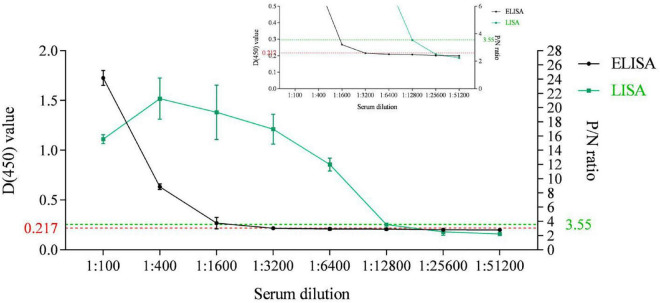
Sensitivity comparison between the p35-Luc LISA (green line with squares) and the p35-ELISA kit (black line with dots) for anti-ASFV antibody detection. The ASFV-positive sera are diluted to different folds. The plots of the inset line charts show the signals under high dilutions, with dotted lines as the cutoff values for positive results. Data are shown with means ± SD. Error bars represent the standard deviations from triplicates.

An indirect ELISA based on the recombinant ASFV p35 protein has been previously established for anti-ASFV antibody detection in our lab (Shi et al., 2021). In the present study, the sensitivity of the p35-Luc LISA was displayed through comparing the sensitivity between the p35 Luc-LISA and the p35-ELISA targeted on an inactivated ASFV-positive and a standard negative control serum. As shown in Figure 5, the p35-Luc LISA could give positive results after the sera were diluted up to 6,400 times, while the p35-ELISA kit could detect them up to dilutions of 1,600 times, suggesting the p35-Luc LISA assay is 4 times more sensitive than the p35-ELISA kit.

### The p35-Luc LISA had good performance in the stability

The performance of the p35-Luc LISA using the antigens prepared in batch to batch was investigated. The reproducibility of the p35-Luc LISA showed that the *CV* values of the p35-Luc LISA in intra-assay was 2.87% to 13.99%, and the *CV* values of the p35-Luc LISA in inter-assay was 0.15% to 12.84% (Table 1), the *CV* values in intra- and inter- reproducibility of the p35-Luc LISA were both less than 15%, which is located in a reasonable range of variation (Li et al., 2018), suggesting the good performance in the reproducibility of the p35-Luc LISA.

**TABLE 1:** Stability of the p35-Luc LISA.

Dilution ratio	p35 In-batch			p35 Batch-to-batch		
	Average value	Standard deviation	CV (%)	Average value	Standard deviation	CV (%)
1:100	15.5526	0.4474	2.88	15.0620	0.6938	4.61
1:400	21.2563	2.9080	13.68	21.0210	0.3327	1.58
1:1600	19.3394	2.7061	13.99	19.4879	0.2100	1.08
1:3200	16.9609	1.4836	8.75	16.1992	1.0772	6.65
1:6400	11.9792	0.6458	5.39	12.5484	0.8050	6.42
1:12800	3.5341	0.1130	3.20	3.5302	0.0055	0.16
1:25600	2.5028	0.3153	12.60	2.7060	0.2873	10.62
1:51200	2.1333	0.2000	9.28	1.8414	0.2365	12.85

#### The agreement between the p35-Luc LISA and commercial ingezim p72-ELISA in detecting anti-ASFV antibodies

As compared with the commercial Ingezim p72-ELISA kit, the reliability of the p35-Luc LISA was evaluated in large-scale investigating field serum samples. As shown in Table 2, the high agreement (97.6%, kappa = 0.9753) between the both methods was observed, and the p35-Luc LISA assay showed a relative sensitivity of 98.44% (95% CI: 0.9447, 99.81%) and a relative specificity of 96.67% (95% CI: 0.9169, 99.80%).

**TABLE 2:** Sensitivity and specificity of the p35-Luc LISA assay for detecting swine serum samples.

		P35-Luc LISA		
		Positive	Negative	Total
Ingezim P72-ELISA	Positive	126	2	128
	Negative	4	116	120
	Total	130	118	248
	Sensitivity (%) (95% CI)	98.44% (0.9447 to 0.9981)		
	Specificity (%) (95% CI)	96.67% (0.9169 to 0.9908)		

## Discussion

Given the pathogenic characteristics of ASFV, a rapid and sensitive diagnostic method is crucial for timely detection and eradication of ASF in affected farms (Wang X. et al., 2020; Daigle et al., 2021). In this study, an improved LISA based on the p35-Luc protein was developed for ultrasensitive detection of anti-ASFV antibodies.

Previous studies showed that the diagnostic method based on LISA exhibit superior characteristics, including rapid, ultrasensitive, high throughput, and easy operation (Wang et al., 2019; Wang T. et al., 2020; Liang et al., 2021; Li et al., 2022). LISA used the cell lysates containing the recombinant protein as diagnostic antigens without the requirement of antigen purification, which will facilitate in rapidly establishing diagnostic methods (Wang X. et al., 2020). As far as the reactive specificity of the p35-Luc protein with the inactivated ASFV-positive sera is concerned, the p35-Luc LISA exhibited high specificity for anti-ASFV sera, while no cross-reactions with the sera raised against other four swine pathogens including PRRSV, CSFV, PCV2 and PEDV was observed, suggesting that the p35-Luc LISA can be used for serological survey of ASFV infection.

NanoLuc^®^ luciferase used in a luciferase-immunoprecipitation system was approximately 150-fold brighter than firefly or Renilla luciferases at equivalent concentrations, the signal of the antigen-antibody reaction can be amplified and better displayed, in addition, the antigens obtained in the eukaryotic expression system might expose more epitopes to be recognized by the corresponding antibody, therefore, LISA was confirmed to be an ultrasensitive diagnostic method as compared to the normal ELISA method (Ludolfs et al., 2009; Wang et al., 2019; Wang X. et al., 2020; Liang et al., 2021; Li et al., 2022), which was also verified in the sensitivity of p35-Luc LISA. In the present study, the detection limit of the p35-Luc LISA assay is 4 times higher than that of the p35-ELISA method previously established in our lab, implying that the p35-Luc LISA could be applied in the early screening and diagnosis of ASF in pig farms.

Lastly, the p35-Luc LISA had a good performance in the reproducibility and reliability. The good agreement (97.6%, kappa = 0.9753) between the p35-Luc LISA and the commercial p72-ELISA kit in investigating 248 field serum samples was observed, suggesting that the p35-Luc LISA is a reliable diagnostic method for detecting anti-ASFV antibody. However, the inconsistent results obtained by the both assays maybe attributed to the difference on the sensitivity of the both assays. In addition, the partial ASFV-negative serum samples included in the present study have been preserved several years, which may increase the possibility of the false-positive results in this parallel assay.

Exception for the reliability of the diagnostic method, the productive cost of the diagnostic kit is one of determinants whether the method is suitable for large-scale testing in the clinical practice. The normal LISA method needs immobilizing the testing serum samples with Protein G coated on the Costar 96-well flat-bottomed luminometer plates (Wang et al., 2019; Wang T. et al., 2020; Liang et al., 2021; Li et al., 2022), in the present study, an improved LISA protocol was provided, the testing serum samples were directly coated with carbonate buffer (pH 9.6), and the improvement was confirmed to be feasible and reliable for detecting anti-ASFV antibodies. The improved protocol greatly simplifies the operation procedure and reduces the productive cost as compared to the normal LISA assay. In addition, it is worth to be noted that the amount of crude antigens obtained in the 2 × 10^5^ 293T cells transfected with 1 μg pNLF-1N-p35 recombinant plasmids could meet the antigens required to detect 1,000 serum samples. Altogether, the present study provided an improved p35-Luc LISA for large-scale serological survey of ASF in pig farms, which is a rapid, ultrasensitive, cost-effective and reliable diagnostic method.

## Data availability statement

The raw data supporting the conclusions of this article will be made available by the authors, without undue reservation.

## Author contributions

ZT: conceptualization, methodology, drafted the article and visualization, supervision, project administration, and funding acquisition. QW: performing the experiments, data curation, validation, and writing the original draft. JY and QN: collecting swine serum samples from pig farms. HZ: data curation and validation. SG, JD, GG, and HY: technical assistance and editing the original draft. All authors contributed to the article and approved the submitted version.
